# Chronic antidepressant treatment rescues abnormally reduced REM sleep theta power in socially defeated rats

**DOI:** 10.1038/s41598-021-96094-0

**Published:** 2021-08-18

**Authors:** Yoshiki Matsuda, Nobuyuki Ozawa, Takiko Shinozaki, Kazuhisa Aoki, Naomi Nihonmatsu-Kikuchi, Toshikazu Shinba, Yoshitaka Tatebayashi

**Affiliations:** 1 Affective Disorders Research Project, Tokyo Metropolitan Institute of Medical Science, 2-1-6 Kamikitazawa, Setagaya, Tokyo, 156-8605 Japan; 2 Department of Psychiatry, Shizuoka Saiseikai General Hospital, 1-1-1 Oshika, Suruga-ku, Shizuoka, 422-8527 Japan

**Keywords:** Depression, Post-traumatic stress disorder, Experimental models of disease, Translational research, Sleep disorders, Neurophysiology

## Abstract

The effects of chronic antidepressant (AD) treatment on sleep disturbances in rodent chronic stress models have not been thoroughly investigated. Here, we show that chronic social defeat stress (SDS) in rats induces prolonged social avoidance, alterations in sleep architecture (increased total rapid eye movement [REM] sleep duration, bout, and shortened REM latency), and contextual but not cued fear memory deficits, even 1 month after the last SDS. These abnormalities were associated with changes in electroencephalography (EEG) spectral powers, including reduced REM sleep theta power during the light phase. Chronic treatment with two different classes of antidepressants (ADs), imipramine and fluoxetine, significantly ameliorated these behavioral, sleep, and EEG abnormalities. Interestingly, REM theta power was normalized by chronic (1 month) but not 1 week AD administration and solely correlated with the ratio (an objective indicator) of social interaction 1 month after the last SDS. These data suggest that reductions in REM sleep theta power, an EEG parameter that has never been directly investigated in humans, is a core sleep symptom in socially defeated rats, and, potentially, also in patients with stress-related psychiatric disorders, including major depressive and posttraumatic stress disorders.

## Introduction

Psychological stressors generally have prominent effects on sleep, especially rapid eye movement (REM) sleep. Disturbed sleep is a prominent and perhaps, even the hallmark feature of stress-related psychiatric disorders, including major depressive disorder (MDD)^1,2^ and posttraumatic stress disorder (PTSD) (for review, see^3^). The most common sleep polysomnography (PSG) abnormalities in patients with MDD include decreased REM sleep latency (i.e., the interval between sleep onset and the first REM sleep period^4^), increased REM density (i.e., frequency of rapid eye movements per REM period), increased REM and decreased non-REM (NREM) sleep durations, and increased REM sleep bouts^2,5,6^. For PTSD, the most recent meta-analysis of 31 PSG studies with approximately 1000 patients found that this disorder is associated with less total sleep time, more disruptions in sleep, and less deep sleep compared with those in controls^7^. Although the meta-analysis failed to find significant overall differences in REM sleep, patients aged 30 years and younger were found to spend a smaller proportion of time in REM sleep than age-matched controls. Comparisons of MDD and PTSD, however, have provided no definitive conclusions about differences in PSG data between the two disorders^8–10^.

Virtually all antidepressants (ADs) suppress REM sleep^2,6^. This effect, likely mediated by norepinephrine (NE) and/or serotonin (5-hydroxytryptamine [5-HT])^11^, is rapid^12^ and continuous for at least 4–8 weeks^13,14^. Both NE and 5-HT systems are wake-active and form part of the ascending arousal system, promoting wakefulness^11^. However, a recent study demonstrated that the 5-HT system, especially its tonic stimulation, promotes sleep by generating homeostatic sleep pressure during wakefulness^15^. Thus, the actual role of 5-HT in sleep, particularly the tonic 5-HT stimulation elicited by chronic AD treatment^16^, remains elusive.

Animal models are critical for understanding the causes, mechanisms, and potential treatments for stress-related psychiatric disorders, as well as the underlying mechanisms of action of ADs. Mouse social defeat stress (SDS) models, especially those developed by Berton et al.^17^ and Krishnan et al.^18^, have been shown to have etiological, predictive, discriminative, and face validity^17–20^. In these models, male C57BL/6J mice are exposed to daily bouts of social defeat by larger, physically aggressive CD-1 mice (5–10 min). The defeated mice are continuously subjected to this psychological stress by sensory interactions with aggressors through clear, perforated dividers in shared home cages for the duration of the experiment (generally 10 days). Although this procedure has been shown to lead to the development of MDD- and anxiety-like behaviors in all C57BL/6J mice, only a subset of mice (called “susceptible” mice, defined by the presence of social avoidance against aggressive CD-1 mice^18^) exhibit maladaptive changes, including near-permanent social avoidance against nonaggressive C57BL6/J littermates^17,21^, anhedonia, and metabolic syndrome^18,22^, all of which can be reversed by chronic but not acute administration of two different classes of ADs, fluoxetine (FLU) and imipramine (IMI)^17,21,23^.

Little is known, however, about any prolonged or maladaptive sleep and/or circadian changes in this mouse model. Dysregulation in the circadian amplitude of body temperatures has been noted in susceptible mice; however, these changes have been shown to naturally disappear 4 weeks after the last SDS^18^. More recently, two independent groups have investigated the effects of SDS on sleep^24,25^; however, most sleep changes were observed only during SDS periods. Furthermore, limited information has been reported about long-term, post-stress effects.

In the present study, we applied Berton–Krishnan-type SDS to rats in order to study post-stress sleep alterations for the first time. We found that SDS induced long-lasting, maladaptive social avoidance linked to sleep alterations for at least 1 month. We then investigated the effects of chronic FLU or IMI treatment on these changes and found that, among several sleep disturbances rescued by ADs, decreased REM sleep theta power was a core sleep symptom associated with maladaptive social avoidance in rats. Finally, impaired contextual but not cued fear memory was observed even 1 month after the last SDS. Based on these data, the effects of REM sleep theta power, an electroencephalography (EEG) parameter that has never been directly investigated in humans, on the pathogenesis of stress-related psychiatric disorders are discussed.

## Results

### Characterization of the rat SDS model

**Figure 1:**
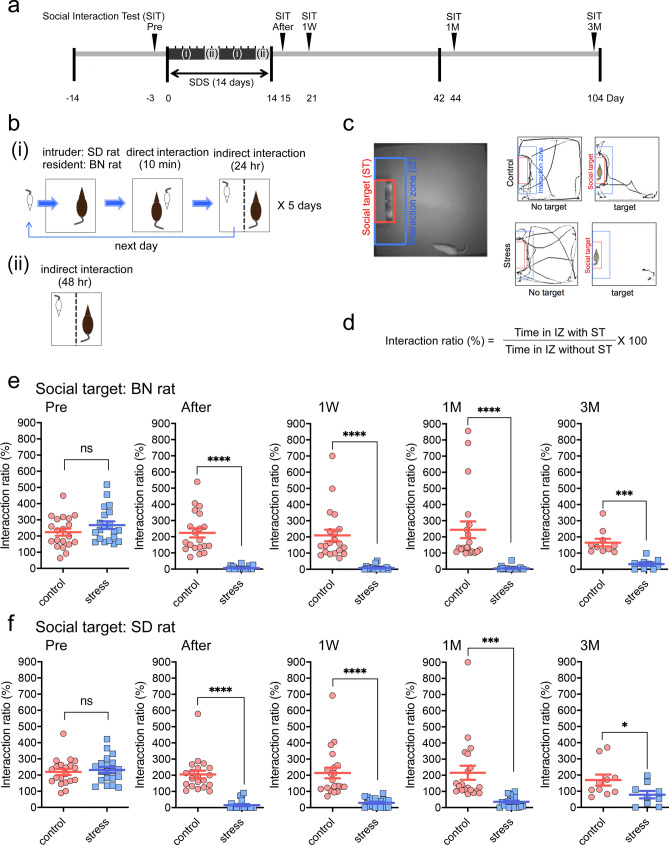
Novel social defeat stress (SDS) rat model. (**a**) Experimental schedules for SDS and the social interaction test (SIT). The 14-day SDS paradigm consisted of two processes ((i) and (ii)). (**b**) The SDS procedure applied to this model: a 10-min direct interaction followed by a 24-h indirect interaction was conducted once per day for five days (i). A 48-h indirect interaction was applied for two days without any direct interaction (ii). This combination was repeated twice during a 14-day SDS period. (**c**) SIT for rats. The interaction zone (IZ) is defined by the blue rectangle. (**d**) Calculation of the interaction ratio. (**e, f**) SIT with BN (**e**) and SD (**f**) rats before (Pre) SDS and at 1 day (After), 1 week (1 W), 1 month (1 M), and 3 months (3 M) after the 14-day SDS. *p < 0.05, ***p < 0.0005, ****p < 0.0001, two-tailed, unpaired *t*-test.

**Figure 2:**
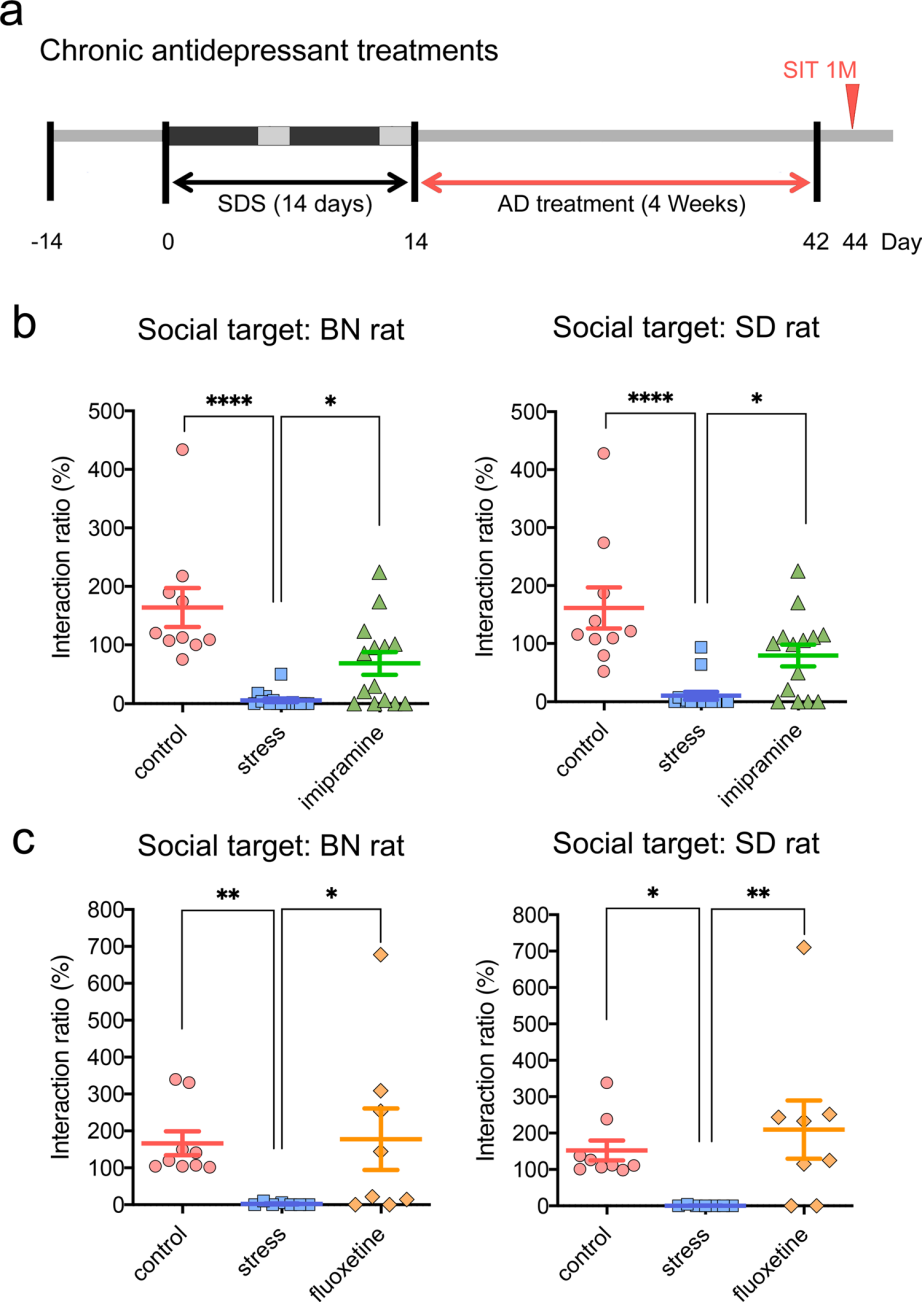
Effects of chronic antidepressant (AD) treatment on social avoidance. (**a**) Experimental schedule for chronic (4 weeks) AD treatment. (**b**, **c**) Social interaction test (SIT) at 1 month after the last social defeat stress (SDS) (Day 44, n = 7–16/group). Note that the AD groups included a certain proportion of nonresponders. *p < 0.05, **p < 0.01, ****p < 0.0001, Kruskal–Wallis test followed by Dunn’s multiple comparisons test.

We adopted the Berton–Krishnan-type SDS paradigm^17,18^ in rats, with some modifications (see "Methods"), employing SD intruder rats and aggressive BN resident rats (Fig. 1a,b). To measure the impact of 14 days of SDS, the social interaction test (SIT) was conducted 1 day after the last SDS (Fig. 1c,d). SD rats exhibited near-complete social avoidance of the unfamiliar resident (BN) rats (Fig. 1e). Furthermore, this near-complete social avoidance was also observed toward unfamiliar, nonaggressive SD littermates (Fig. 1f), suggesting that social avoidance was not simply an adaptive response to SDS but also a maladaptive response^17,21^. This social avoidance of both BN and unfamiliar SD rats persisted for at least 3 months (Fig. 1e,f). Chronic imipramine (IMI) or fluoxetine (FLU) administration for 4 weeks (Fig. 2a) significantly ameliorated this social avoidance, both towards the BN rats (Fig. 2b) and the unfamiliar SD rats (Fig. 2c), though approximately 30% to 40% of test rats showed no improvements (Fig. 2b,c).

Sucrose preference test was also performed one month after the last SDS and no significant difference was found between groups (Supplementary Fig. S1). We, however, found a significant decrease in the sucrose intake amounts in stressed rats, which was rescued by chronic FLU administration.

### Sleep architecture analyses

**Figure 3:**
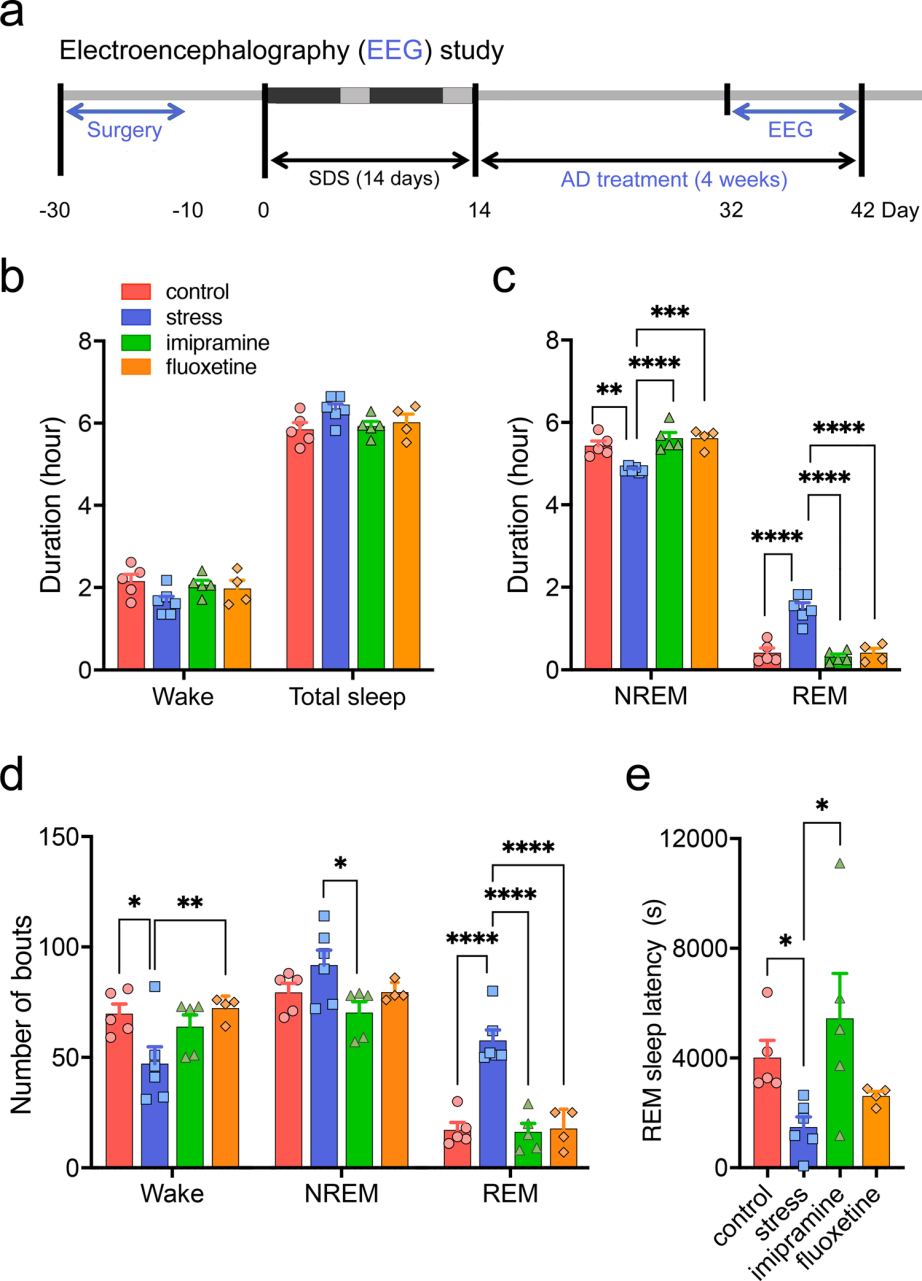
Effects of social defeat stress (SDS) and chronic antidepressant (AD) treatment on sleep architecture. (**a**) Experimental schedule for sleep analysis. (**b**) Durations of wakefulness and total sleep during the light phase (8:00 [ZT0]–16:30 [ZT8.5]) at 1 month after the last SDS. ZT; zeitgeber time. (**c**) Total durations of non-rapid eye movement (NREM) and rapid eye movement (REM) sleep during the light phase. *F*_(3, 32)_ = 41.55, *p* < 0.0001, **p < 0.005, ***p < 0.0005, ****p < 0.0001, two-way (group x sleep stage) repeated analysis of variance (ANOVA) with Tukey’s multiple-comparisons test. n = 4–6/group. (**d**) Total number of bouts of NREM and REM sleep, as well as wakefulness during the light phase (ZT0–ZT8.5). *F*_(6, 48)_ = 10.06, *p* < 0.0001, *p < 0.05, **p < 0.01, ****p < 0.0001, two-way ANOVA with Tukey’s multiple-comparisons test. n = 4–6/group. (**e**) REM sleep latency. *p* = 0.0092, *p < 0.05, Kruskal–Wallis test followed by Dunn’s multiple comparisons test. n = 4–6/group.

Sleep/wake stages were determined using EEG, and skeletal muscle atonia was evaluated using electromyography (EMG) (Fig. 3a, see Supplementary Fig. S2). In rodents, theta oscillations have been shown to occur in all hippocampal subregions during wakefulness and REM sleep^26^; thus, they are the best EEG markers for these states (see Supplementary Fig. S2d). During the first 8.5 h (8:00–16:30) of the light phase (8:00–20:00), we found no significant differences in the durations of the wake and total sleep states between control and SDS rats (Fig. 3b). SDS, however, significantly decreased the duration of NREM sleep and increased the duration of REM sleep (Fig. 3c). SDS also decreased the number of wake bouts and increased the number of REM sleep bouts (Fig. 3d). In addition, we found that SDS significantly shortened the latency to the onset of the first REM period (Fig. 3e) (for the definition of REM sleep latency, see Supplementary Fig. S2e).

Chronic AD treatment restored most sleep alterations to a significant degree (Fig. 3c–e), except for the number of wake bouts and REM sleep latency [IMI failed to restore the former (Fig. 3d) and FLU failed to restore the latter (Fig. 3e)]. We also investigated the time-dependent effects of chronic FLU treatment (see Supplementary Fig. S4); FLU slowly restored NREM duration, while REM duration and latency were restored more rapidly (1 week). FLU decreased the number of REM sleep bouts relatively rapidly (1 week); however, the effects became significant only at 1 month.

### EEG spectral power analysis

**Figure 4:**
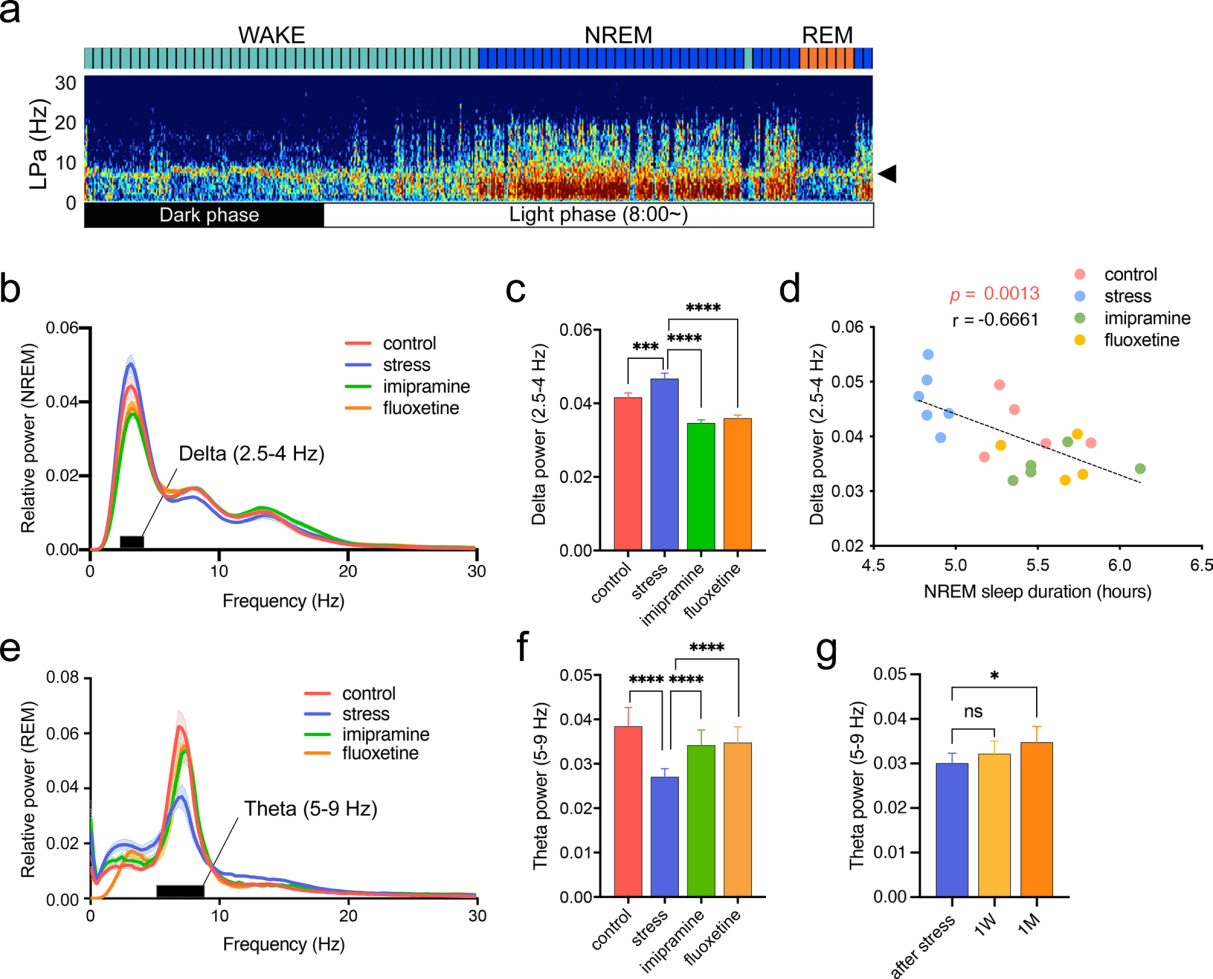
Effects of social defeat stress (SDS) and chronic antidepressant (AD) treatment on electroencephalography (EEG) spectral power. (**a**) A representative spectrogram showing the predominant frequency band at the theta range (arrowhead) during wakefulness and rapid eye movement (REM) sleep (30 s/epoch). (**b**) EEG-relative power spectra during NREM sleep in the light phase (ZT0–ZT8.5). The black bar indicates the selected delta (2.5–4 Hz) band. (**c**) Average relative spectral power of the delta band during NREM sleep. *F*_(5, 96)_ = 5.638, *p* = 0.0001, ***p < 0.0005, ****p < 0.0001, two-way analysis of variance (ANOVA) followed by Tukey’s multiple comparisons test. (**d**) A significant negative correlation between NREM sleep duration during the light phase and the relative delta power (2.5–4 Hz). *p* = 0.0013, *r* = −0.6661. (**e**) Relative power spectra during REM sleep in the light phase. The black bar shows the selected theta (5–9 Hz) band. (**f**) Average relative theta power during REM sleep. *F*_(45, 256)_ = 3.033, *p* < 0.0001, *p < 0.05, **p < 0.005, ****p < 0.0001, two-way ANOVA followed by Tukey’s multiple comparisons test. (**g**) Time-dependent effects of fluoxetine (FLU) on average relative spectral powers during REM sleep. *F*_(15, 144)_ = 18.28, *p* < 0.0001, *p < 0.05, two-way ANOVA followed by Tukey’s multiple comparisons test.

The average EEG spectral powers of NREM and REM sleep during the first 8.5 h (8:00–16:30) of the light phase (8:00–20:00) were analyzed (Fig. 4a). We found that SDS increased the average relative delta power during NREM sleep, while IMI and FLU treatment decreased it (Fig. 4b,c). This NREM delta power correlated significantly with NREM duration (Fig. 4d) but not with total sleep duration (see Supplementary Fig. S3a) during the first 8.5 h (8:00–16:30) of the light phase. Almost the same results were obtained even when the first 10 epochs of NREM delta power was introduced instead of those during the first 8.5 h (8:00–16:30) of the light phase (8:00–20:00) (Supplementary Fig. S3b-d). Since the spectral power of delta oscillations (2.5–4 Hz) during NREM sleep is regarded as one of the best markers for homeostatic sleep-need levels^27,28^, our data suggest that SDS increased the homeostatic sleep need in rats, while ADs decreased it. Surprisingly, during REM sleep, SDS significantly decreased the average relative REM sleep theta power (5–9 Hz), while chronic AD treatment increased it (Fig. 4e,f). Furthermore, chronic FLU treatment increased REM sleep theta power in a time-dependent manner (Fig. 4g, see Supplementary Fig. S4).

### Correlation between EEG sleep parameters and social interaction ratios

**Figure 5:**
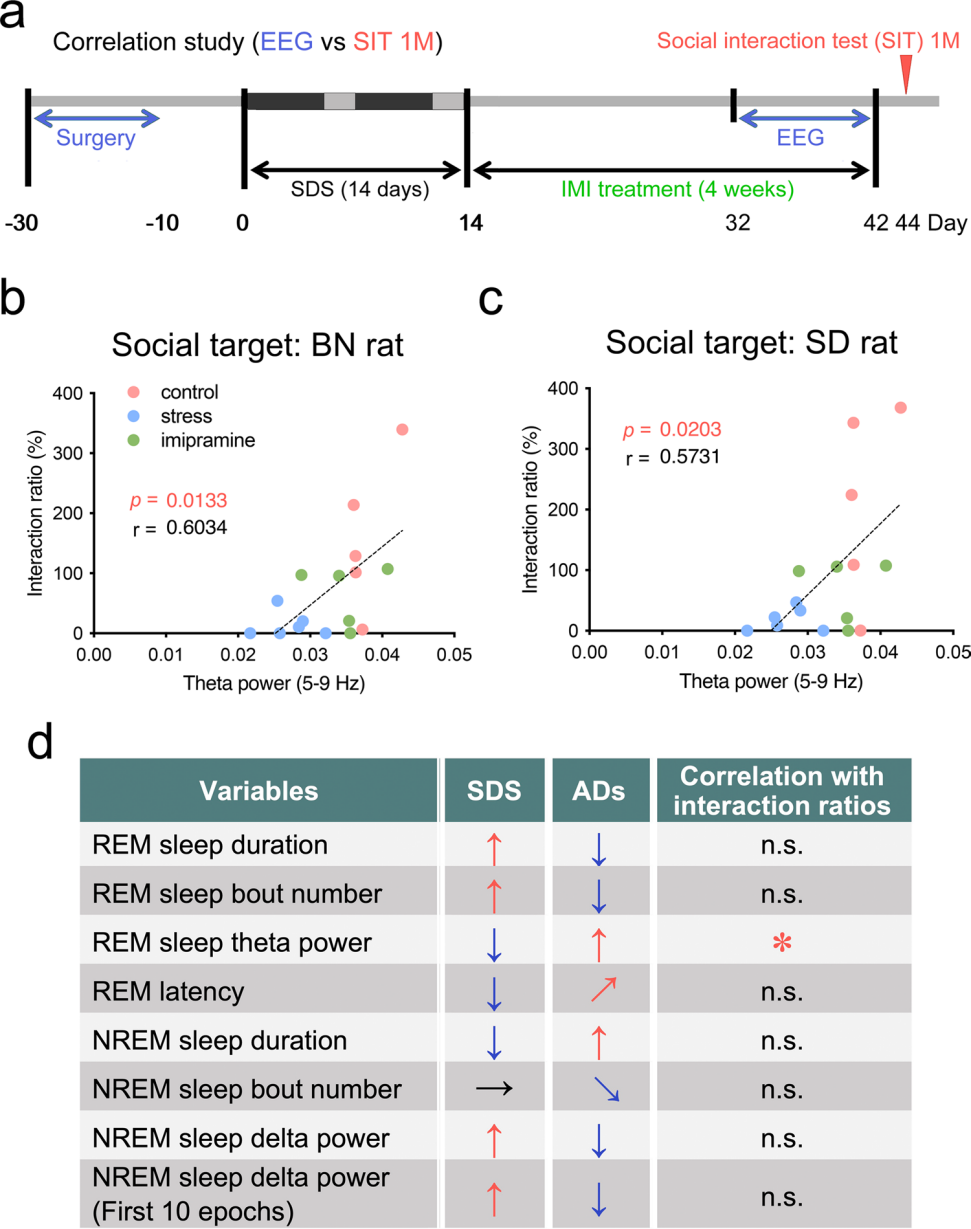
Correlations between the rapid eye movement (REM) sleep theta power and interaction ratios 1 month after the last social defeat stress (SDS). (**a**) Experimental schedule for correlation analysis. (**b**, **c**) Correlations between the average relative REM theta power during the light phase (ZT0–ZT8.5) and interaction ratios with social targets (BN rats; **b**, SD rat; **c**) 1 month after the last SDS. (**d**) Summary of the correlation study (see Supplementary Fig. S5). Note that, among the various sleep variables, only the REM sleep theta power significantly correlated with the social interaction ratios (*).

To evaluate which sleep parameters were responsible for the observed maladaptive social avoidance in our rat model, we performed correlation analysis between social interaction ratios at 1 month and sleep variables (REM and NREM sleep durations and bouts, REM latency, REM theta power, and NREM delta power) (Fig. 5a–d). In these analyses, we analyzed the data from all the experimental groups together (a total of 16 SD rats including controls (n = 5), stressed (n = 6), and stressed rats with IMI treatment (n = 5)). We found that the interaction ratios toward BN and unfamiliar SD rats at 1 month after the last SDS correlated significantly with the relative average REM sleep theta power (BN: *r* = 0.6034, *p* = 0.0133, Fig. 5b; SD: *r* = 0.5731, *p* = 0.0203, Fig. 5c). No significant correlations were found between interaction ratios and REM or NREM durations or bout numbers, REM latency, or NREM delta power both during the light phase (8:00–16:30) and the first 10 epochs (Fig. 5d, see Supplementary Fig. S5).

### Fear-conditioning analysis

**Figure 6:**
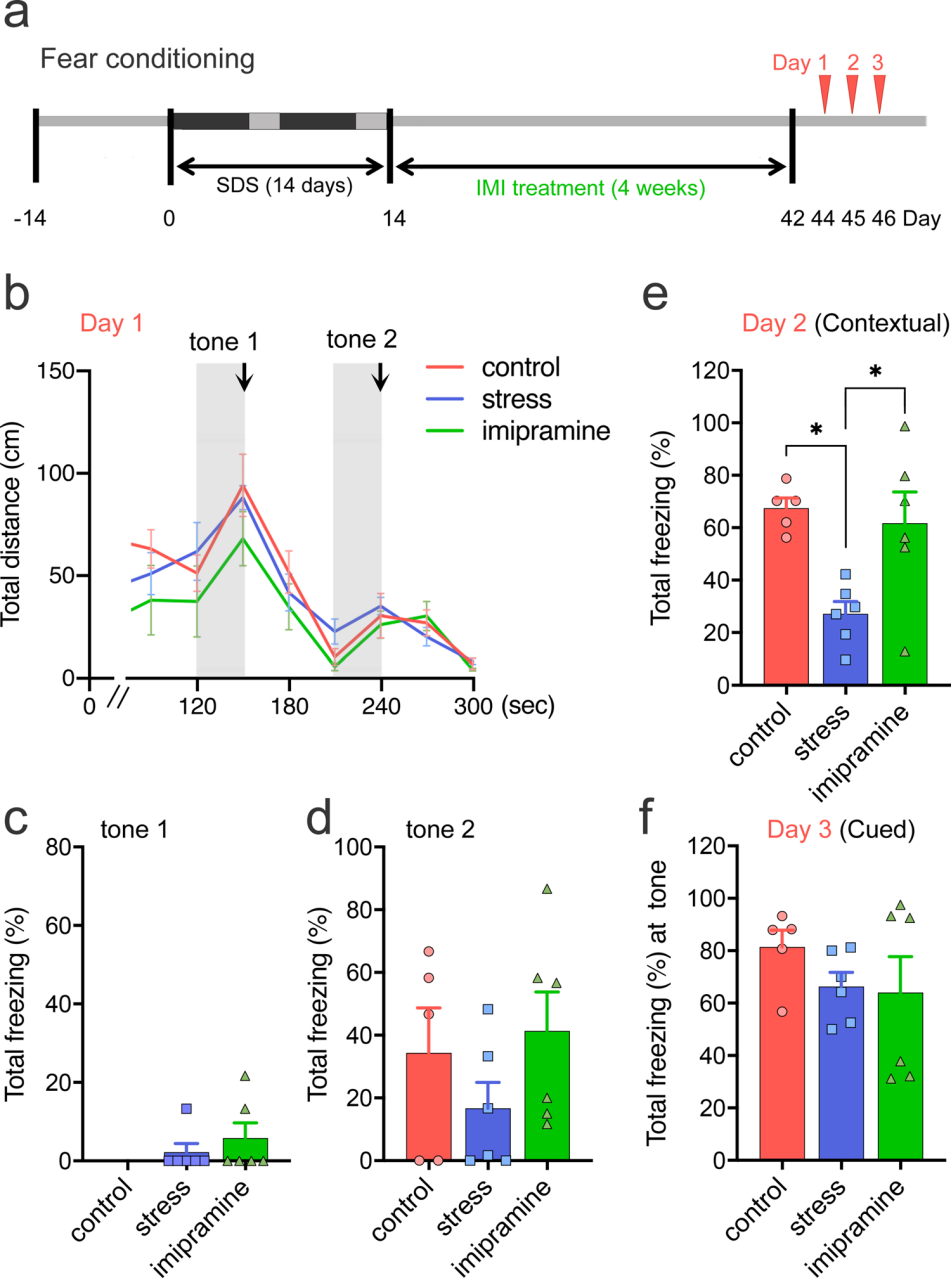
Memory function in a novel social defeat stress (SDS) rat model. (**a**) Experimental schedule for the fear-conditioning test. (**b**–**f**) Fear-conditioning test at 1 month after the last SDS (Days 44–46). Arrows indicate a 2 s period of delivery of 0.3 mA current. On Day 1, no group differences were found in the total distance moved (**b**) or the total freezing during tones 1 (**c**) or 2 (**d**). Contextual recall memory test on Day 2 (**e**). Recall impairment was found in the stressed rats, which was recovered by imipramine (IMI) treatment. *p < 0.05, one-way ANOVA followed by Tukey’s test. Cued recall memory test on Day 3 (**f**). n = 5–6/group.

We then examined how disturbed sleep affects fear-conditioned contextual and cued memory formation in our model at 1 month (on Days 44–46; Fig. 6a). Fear conditioning is a single-trial, associative learning task in which an animal learns to fear a new environment (context) or a discrete conditioned stimulus (cue), such as white noise, because of an association between these conditioned stimuli and an aversive unconditioned stimulus, e.g., a foot shock (for review, see^29^). When exposed to the same context or cue at 1 or 2 days after training, animals exhibit a variety of fear responses, including freezing behavior^30^. While both contextual and cued learning are amygdala-dependent, contextual learning is also hippocampus-dependent (for review, see^31^). During conditioning (Day 1), freezing behavior between the groups was not significantly different in context A (Fig. 6b) or during tones (Fig. 6c,d). On Day 2, SDS significantly reduced freezing behavior (Fig. 6e) in context A, suggesting that our SDS paradigm induced a contextual memory deficit even 1 month after the last SDS. Chronic IMI treatment significantly rescued this deficit (Fig. 6e). On Day 3, the rats were placed in a novel context (context B) for a total of 5 min for cued recall testing. Freezing behavior during the tone (2 min) did not differ between the groups, with each showing a robust and selective freezing response based on the cue (tone) (Fig. 6f).

## Discussion

In the present study, we applied the Berton–Krishnan-type SDS paradigm^17,18,21^ to rats for 14 days and found that SDS induced prolonged and maladaptive social avoidance in association with sleep alterations even at 1 month after the last SDS, with restoration of sleep alterations after chronic treatment with two different classes of ADs, IMI and FLU. In addition, we found that, among various sleep parameters, REM sleep theta power was the only variable that correlated with the social interaction ratio at 1 month. Finally, impairments in contextual but not cued fear memory were also found at 1 month. These behavioral responses to SDS and chronic AD treatment in rats were somewhat different from those that have been previously reported in the mouse SDS model.

First, in contrast to the mouse SDS model in which the SIT distinguishes “susceptible” from “resilient” mice after SDS^18,21^, a 14-day SDS in rats induced near-complete social avoidance even against nonaggressive SD littermates (Fig. 1f), suggesting that all test rats became susceptible in our model. Furthermore, chronic AD treatment rescued impaired social interactions significantly, though some nonresponders persisted in our model (Fig. 2b,c). As far as we know, no such evidence of nonresponders has been reported in the mouse SDS model.

It is well-known that approximately one-third of MDD patients fail to respond to conventional AD therapies^32^. Thus, if social avoidance against nonaggressive SD littermates (Fig. 1f) is a maladaptive symptom recapitulating certain aspects of stress-related psychiatric disorders, such as MDD or PTSD, the identification of stressed rats who failed to respond to chronic AD treatment during SIT (Fig. 2) may have a translational value. The idea that social avoidance can distinguish “susceptible” from “resilient” animals was first proposed in the mouse SDS model^18^. In this model, “susceptible” mice, as classified by social avoidance of aggressive CD-1 mice, also exhibited maladaptive social avoidance against nonaggressive C57BL6/J littermates^17,21^, anhedonia, and metabolic syndrome, which are all highly correlated with one another^17–19,21,22^. Furthermore, only chronic but not acute AD administration improved social interactions in defeated mice^17^. Thus, it is plausible that the social avoidance of nonaggressive SD littermates observed in our study was not just an adaptive symptom of agoraphobia or social phobia disorder but a kind of maladaptive symptom related to MDD and/or PTSD, which could only be rescued by chronic AD treatment.

Second, we found that chronic SDS induced disturbed sleep for up to 1 month, which was mostly rescued by chronic AD (IMI and FLU) treatment (Fig. 3). Notably, among several sleep parameters, REM sleep theta power was the only variable that correlated with social interaction ratios at 1 month (Fig. 5 and Supplementary Fig. S5). In contrast, NREM sleep delta power (= slow-wave activity) both during the first 8.5 h (8:00–16:30) of the light phase (8:00–20:00) or the first 10 epochs (300 s) was correlated with NREM sleep (Fig. 4d) but not with total sleep duration (See Supplementary Fig. S3a) or social interaction ratios at 1 month (Fig. 5 and Supplementary Fig. S5), suggesting that, while NREM sleep delta power is a marker of sleep homeostasis^27,28^, it may be independent of maladaptive social avoidance.

Little is known about the post-stress features of sleep architecture in rodent chronic stress models (see Supplementary Table S1). For example, a pioneering study by Moreau et al.^33^ first investigated the effects of unpredictable chronic mild stress (UCMS) on sleep in male albino rats, finding a reduced latency until the first REM sleep episode and an increased time spent in REM sleep during the second week of a 3-week stress regimen, with progressive disappearance of these findings after the termination of stress. Subsequent studies using UCMS have shown similar sleep changes occurring only during stress periods (see Supplementary Table S1). A study by Hegde et al. first investigated the post-stress effects of 2-h chronic immobilization stress (CIS) for 10 days, finding that a subgroup of stressed rats showed an increased REM sleep duration in association with attenuated REM sleep theta power even 3 weeks after the end of stress^34^.

In rodent SDS models, most studies have used the method of Miczek et al. (1979)^35^, which involves 3–5 min (or less than 10 times) of direct social interaction (or conflicts) combined with 0.5–1 h (but not 24 h) of indirect interaction. In early studies, the effects of single or double SDS on the subsequent sleep/wake states were extensively studied. Page et al.^36^ first investigated the effects of repeated Miczek-type SDS for 4 weeks on sleep and found that sleep changes were observed only during the stress period, with a return to normal levels at the end of this period. More recently, Grafe et al.^37^ reported that, when test rats were divided into passive or active coping groups based on their submissive behaviors during direct social conflicts, passive-coping rats continuously showed decreased SWS sleep time even 2 weeks after the end of stress.

Two groups^24,25^ have employed the Berton–Krishnan-type chronic mouse SDS model for sleep studies. In the first study^25^, REM sleep duration, bouts, and theta power (per day) increased during SDS, while REM sleep theta power appeared to decrease on Day 10 of the SDS. All these changes, however, were observed mostly during SDS, with a return to normal levels by 5 days after the last SDS^25^. In the second study^24^, no apparent sleep changes were found at 3 days after a 10-day SDS; however, subsequent sleep deprivation induced a blunted increase in sleep pressure in stressed mice compared to control mice, as measured by slow-wave activity during NREM sleep. None of these studies, however, investigated the long-term, post-stress effects of SDS or the effects of chronic AD treatment on disturbed sleep.

Third, impairments in contextual but not cued fear memory were found at 1 month after the last SDS, which was rescued by chronic IMI treatment (Fig. 6e,f). In the mouse SDS model, however, the effects of SDS on fear learning have been inconsistent^38^, with susceptible mice typically showing increased contextual and cued fear learning^39^. However, there have also been reports of decreased fear learning^40^, increased fear learning in resilient mice only^41^, and no effects of SDS on either group^42^. Interestingly, similar contextual but not cued fear memory deficits were observed in rodents with prolonged (72–96 h) REM sleep deprivation (SD) before conditioning^43,44^ or in rodents with post-conditioning REM SD^45^. Consistently, optogenetic silencing of γ-aminobutyric acid–releasing (GABA) neurons in the medial septal area (MS) (MS-GABA) during post-conditioning REM sleep has been shown to result in contextual (but not cued) fear memory deficits^46^.

Cognitive dysfunction is a common feature of MDD, contributing to a serious decline in the patient’s quality of life^47,48^. Patients with MDD typically show impaired recollection, poor memory for positive events, potentiated memory for negative events, and “overgeneral” autobiographical retrieval, probably due to an inability to retrieve the precise context (for review^48^). These features may have been partially recapitulated in our model, in which the amygdala-dependent cued learning was intact, while the hippocampus-dependent contextual fear learning was impaired (Fig. 6). We speculate that disturbed sleep, especially reduced REM sleep theta power but not increased REM sleep duration (Fig. 3c), caused impaired contextual fear learning. Future studies are warranted to prove this hypothesis in both rodents and humans.

REM sleep theta power may also play an important role in the pathogenesis of some symptoms of stress-related psychiatric disorders. Although the generation of hippocampal theta oscillations requires an intact MS^49^, REM sleep generation itself does not require the MS^50,51^. Similar independent regulation between REM sleep theta generation and timing have been demonstrated by Boyce et al.^46^, in which silencing of MS-GABA during REM sleep reduced REM sleep theta power but did not perturb sleeping behavior or the probability of sleep state transitions. Similarly, we found that chronic FLU treatment chronically increased REM sleep theta power in a time-dependent manner (Fig. 4g, see Supplementary Fig. S4), which contrasted strikingly to the relatively rapid (1 week) effects of FLU on REM duration and latency. These data suggest that AD treatment chronically changes brain areas regulating REM sleep theta power (e.g., MS, hippocampus, and septohippocampal pathway), while more rapidly affecting brain areas regulating REM sleep timing (e.g., brainstem, midbrain, and hypothalamus) (see review^52^), although both areas were damaged by SDS.

ADs produce a rapid increase in extracellular levels of 5HT and NA; however, the onset of an appreciable clinical effect usually takes at least 3–4 weeks^53^. This delay has long been suggested to demonstrate that slow neurochemical and structural changes take place within monoaminergic-projecting limbic areas, including both MS^54,55^ and the hippocampus^55,56^. One such example is adult dentate gyrus (DG) neurogenesis in the hippocampus^57^. REM sleep theta power may be another potential candidate, as demonstrated in the present study, although almost nothing is known about REM sleep theta power in humans.

The relationship between DG neurogenesis and REM sleep theta power is unclear. For example, recent studies have revealed that chronic stress models are associated with a decrease in neurogenesis in the ventral but not the dorsal DG, while chronic AD treatment increases neurogenesis predominantly in the ventral DG^58^. In the present study, we measured REM theta oscillations from the dorsal side but not the ventral side of the hippocampus (see Supplementary Fig. S2), suggesting that unidentified slow changes, rather than DG neurogenesis, may take place in the MS, dorsal hippocampus, and their pathways, both during SDS and chronic AD treatment in rats.

There are additional important implications of this study. For example, the role of REM sleep in memory consolidation has long been unclear because marked suppression of REM sleep (durations) in subjects treated with ADs produces no detrimental effects on cognition^59,60^ However, this argument could be reconsidered if REM sleep theta power is a more essential factor for memory consolidation and AD treatment (as demonstrated in the present study) than REM sleep duration. Interestingly, Grosmark et al.^61^ found an overall reduction in the firing rates of the hippocampus within triplets of NREM-REM-NREM cycles, together with an increased firing synchronized with ripples in NREM sleep, possibly reflecting memory replay^62^. Notably, both firing rate reductions and synchronization with ripples in NREM sleep were highly correlated with theta power in the interleaving REM sleep episode, suggesting a prominent role of REM sleep theta power in sleep-related neuronal plasticity. More recently, Swift et al.^63^ found an interesting link between the locus coeruleus (LC) NA activity, REM theta power, and memory consolidation. LC activity normally decreases to near-zero firing rate during spindle-rich NREM-intermediate sleep as well as REM sleep. They found that optogenetically enhanced LC activity during sleep reduces the rate of spindle occurrence, REM theta power, and NREM delta power in correlation with hampered spatial memory consolidation^63^, suggesting that insufficient silencing of the LC activity during REM sleep may impair sleep-related brain plasticity. Such research direction is also warranted in the future.

The limitations of this study should be noted. First, we failed to provide clear positive data related to anhedonia, resulting in the failure of a clear-cut discussion of the nature of this model. We performed a sucrose preference test; however, the data have been unclear thus far (Supplementary Fig. S1). Second, from the standpoint of disturbed sleep, we could not conclude if our model was appropriate for MDD, PTSD, or both, since no single sleep variable has been found to reliably distinguish patients with MDD or PTSD from healthy controls^8–10^. Third, REM density, one of the most reliable sleep changes observed in patients with MDD, could not be measured in our paradigm. Fourth, we did not include experimental groups (control + AD) in the current study, partly because many such studies have already been reported since the 1950s (e.g., Gao et al.^64^, and the only likely conclusion is that ADs suppress REM sleep time. Finally, all data in the current study were correlational but not causal in nature.

In conclusion, we have established a novel rat SDS model for stress-related psychiatric disorders, including MDD and PTSD, and have provided valid evidence related not only to behavioral effects but also to sleep architecture. We speculate that the septohippocampal pathway, including the MS and hippocampus, may be partially or largely impaired by SDS, resulting in both emotional and/or cognitive symptoms in our model. Our EEG analysis demonstrated that chronic AD treatment restored maladaptive social avoidance, potentially by improving REM sleep theta power over the long term. Although further validation in humans is required, our model may have utility for the development of new pharmacotherapies for MDD, PTSD, and the sleep disturbances associated with them.

## Methods

### Animal care and use

All animals were treated according to the protocols and guidelines approved by the Animal Use and Care Committee of the Tokyo Metropolitan Institute of Medical Science and the ARRIVE guidelines. All animals were kept under standard laboratory conditions (12 h light/dark [LD] cycle [LD12:12]; lights on at 8:00 a.m. and off at 8:00 p.m. with food and water available ad libitum, unless otherwise indicated). A total of 105 SD rats (control = 39; stressed = 40; ADs = 23; social target = 3) and 46 Brown Norway (BN) rats (aggressor = 42; social target = 3) were used for the experiments.

### SDS

A repeated SDS paradigm, originally developed in mice^17–20^, was applied to rats, with elongation of the SDS period from 10 to 14 days (see below). Briefly, male SD rats (5–6 weeks old at arrival and housed individually, 8 weeks old at the onset of stress) were transferred into the home cage of a male BN rat (retired breeder, > 7 months old; all rats from Charles River Laboratories Japan, Inc., Yokohama, Japan) for 10 min (Fig. 1a,b). During this direct contact period, submissive behaviors, including a supine posture, were generally observed in SD rats, signifying that they had assumed a subordinate position. If the resident BN rat did not initiate an attack within 5 min or if the intruder SD rat did not exhibit any submissive behaviors, the SD rat was transferred into a new resident’s home cage. Other important aspects of the direct contact period included avoidance of severe injuries and damage to the EEG/EMG electrodes, if applicable. After 10 min of direct contact, the resident and intruder rats were kept in indirect contact for 24 h using a perforated clear vinyl chloride divider in the resident cage (see Supplementary Fig. S1). The intruders were then confronted with new residents daily (Fig. 1a,b) for 5 days, after which the rats were kept in indirect contact for 2 days, over the weekend (Fig. 1a,b). This process was repeated the following week for a total of 14 days (Fig. 1a). Control animals were housed on one side of a perforated divider without a resident. All rats were housed individually, except during the SDS period.

### Chronic AD treatment

After the 14-day SDS paradigm, a tricyclic AD, IMI (20 mg/kg/day, [ICN, Aurora, OH, USA]), a selective serotonin reuptake inhibitor, FLU (5 mg/kg/day [Cayman, MI, USA])^17,65^, or saline was intraperitoneally administered to SDS rats between 14:00–16:00 for 4 weeks. Saline was intraperitoneally administered to control rats for 4 weeks. Of note, IMI and its metabolite, desipramine, inhibit both serotonin and norepinephrine reuptake, while FLU inhibits serotonin reuptake.

### SITs

An experimental SD rat was placed inside an open field (90 × 90 × 45 cm, 30 lx), and movement was monitored for three consecutive sessions of 2.5 min each, with approximately 5-min intervals. During the first session (“no target”), an empty wire mesh cage (30 × 15 × 15 cm) was placed at one end of the field (Fig. 1c). During the second and third sessions (“target”), an unfamiliar BN retired breeder rat (second) and an SD littermate (third) were placed in the mesh cage (Fig. 1c). Locomotor activity measurements (distance traveled) and time spent in the interaction zone were quantified using custom applications (IMAGE EP and IMAGE FZ, O'hara & Co., Ltd., Tokyo, Japan) developed by the public domain NIH IMAGE program (NIH, Bethesda, USA). The social interaction ratio (%) was defined by the following formula: (time (s) spent in the interaction zone (IZ) with social target (ST)/time (s) spent in the IZ without ST) × 100 (Fig. 1d).

### Surgery and EEG recording

Electrode implantation was performed as previously described^66^. Briefly, under pentobarbital sodium (Somunopentyl; Kyoritsu, Tokyo, Japan) anesthesia (60 mg/kg, i.m.), rats were fixed to a stereotaxic apparatus (SR-6M; Narishige, Tokyo, Japan). Nonpolarized Ag/AgCl screw electrodes (1 mm in diameter, O'hara & Co., Ltd., Tokyo, Japan) were then implanted epidurally on the left side of the parietal cortex (LPa; Br -2.0, L1.5) (see Supplementary Fig. S1). The reference and ground electrodes were placed above the cerebellum. An EMG stainless steel electrode surface was subcutaneously placed on the dorsal neck muscle. Lead wires from all electrodes were soldered to a small square socket and mounted on the skull with acrylic resin cement, along with the electrodes. A recovery period of more than 10 days was scheduled prior to the start of the experiment.

To collect EEG data, rats were moved to a cylindrical experimental cage (35-cm diameter) with a soundproof box kept in LD12:12 (see supplementary Fig. S1) and connected via a handmade recording cable with a built-in operational amplifier (TL074; Texas Instruments, Dallas, TX) to lower the cable impedance and reduce electrical and movement artifacts. This stage was recorded for 27 h (3-h habituation and 24-h recording), starting at 13:30 and ending at 16:30 (+ 1 d). Signals were amplified and filtered (gain of 2000 and time constant of 0.1 s for EEG; gain of 5000 and time constant of 0.003 s for EMG; high cut-off filter for both; AB-621G, Nihon-Koden, Tokyo, Japan), digitized at a sampling rate of 500 Hz (Power 1401; Cambridge Electronic Design, Cambridge, UK), and stored using the data acquisition software, Spike2 (Cambridge Electronic Design, Cambridge, UK). Since only two soundproof boxes and two rats could be measured simultaneously, the 27-h EEG measurements were allocated randomly during Days 32–42 to match the average post-stress days among the groups.

### Sleep scoring

Sleep/wake stages were scored using the automatic scoring protocol incorporated in the Spike2 software (rat sleep auto script). These scores were calculated by analyzing EEG and EMG signals during consecutive 30-s epochs. In addition, the accuracy of scoring was confirmed manually (See Supplementary Fig. S1).

### Fear conditioning

On the first day of testing, the animals were placed in a sound-attenuated, clear, acrylic chamber with a grid floor; house lights (20 lx); and background noise provided by a built-in fan (context A) for 5 min. After 2 min of habituation, rats experienced two consecutive tones (white noise, 60 dB) for 30 s, followed by a foot shock of 0.3 mA for 2 s. The interval between the first and second tones was 30 s. On the second day, the animals were tested for contextual memory in the same box (context A) for 5 min, but without tone stimuli. On the third day, the animals were tested for cued memory in the box, now bearing a different appearance (black hollow triangle pole) (context B), but with the tone sounding for a 5-min period. Locomotor activity and freezing behavior were monitored and analyzed, as described above.

### Sucrose preference

The sucrose preference test was evaluated based on the published protocol^18^. This test consisted of a 2-bottle procedure in which rats were given the choice between water and 1% sucrose solution. Rats were habituated to drink water from two bottles over the course of the experiment. On Day 43 after the last stress, one of the bottles was changed to 1% sucrose solution. Water and sucrose intake was measured the following day (44 days). The position of the sucrose bottle was counterbalanced (left versus right) across the different cages to control for potential side-preference bias. Sucrose intake and the preference for sucrose over water (sucrose/[sucrose + water]) were used as measures for sensitivity to reward.

### Statistical analyses

All data are presented as mean ± SEM. All statistical analyses were performed using GraphPad Prism 8 (GraphPad, La Jolla, CA, USA). For statistical comparisons between two groups, the parametric, unpaired *t*-test was used. For datasets comparing more than two groups, the Kruskal–Wallis test, Brown–Forsythe, Welch's ANOVA test, and one- or two-way ANOVA with post hoc comparisons were used. A *p* value < 0.05 was accepted as statistically significant.

## Supplementary Information

Supplementary Information 1.

Supplementary Information 2.

## Data Availability

The data used to support the findings of this work are available from the corresponding author upon reasonable request.
