# Supplementary material for: Chronic antidepressant treatment rescues abnormally reduced REM sleep theta power in socially defeated rats
Source: Sci Rep. 2021 Aug 18;11:16713. doi: 10.1038/s41598-021-96094-0 (PMC8373914; doi:10.1038/s41598-021-96094-0)
Supplement: Supplementary file 1 — Supplementary Information 1. [file 41598_2021_96094_MOESM1_ESM.pdf]

Supplementary Materials for

Chronic antidepressant treatment rescues abnormally reduced REM sleep theta power in socially defeated rats

Yoshiki Matsuda, Ph.D., Nobuyuki Ozawa, Ph.D., Takiko Shinozaki, B.D., Kazuhisa Aoki, Ph.D., Naomi Nihonmatsu-Kikuchi, Ph.D., Toshikazu Shinba, M.D., Ph.D., Yoshitaka Tatebayashi, M.D., Ph.D.

Correspondence to: Yoshitaka Tatebayashi M.D., Ph.D. (tatebayashi-ys@igakuken.or.jp)

This file includes:

Supplementary Figs. S1 to S5

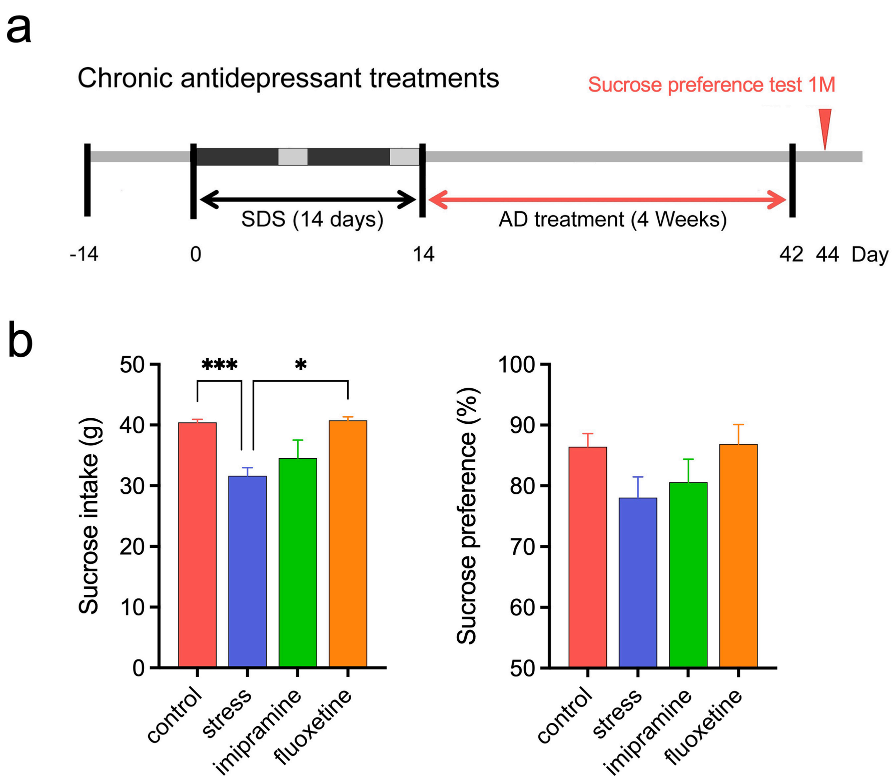

Supplementary Fig. S1 Sucrose preference test for the measurement of social defeat stress (SDS)-induced anhedonia. (**a**) Experimental schedule for sucrose preference test. The stressed rats were treated with antidepressants (ADs) for 4 weeks. (**b**) Sucrose intake and preference test at 1 month after the last SDS. $F_{(3, 59)} = 7.813$, $p = 0.0002$, $*p < 0.05$, $***p < 0.0005$, one-way analysis of variance (ANOVA) followed by Tukey's multiple comparison test for sucrose intake. n = 7–22/group.

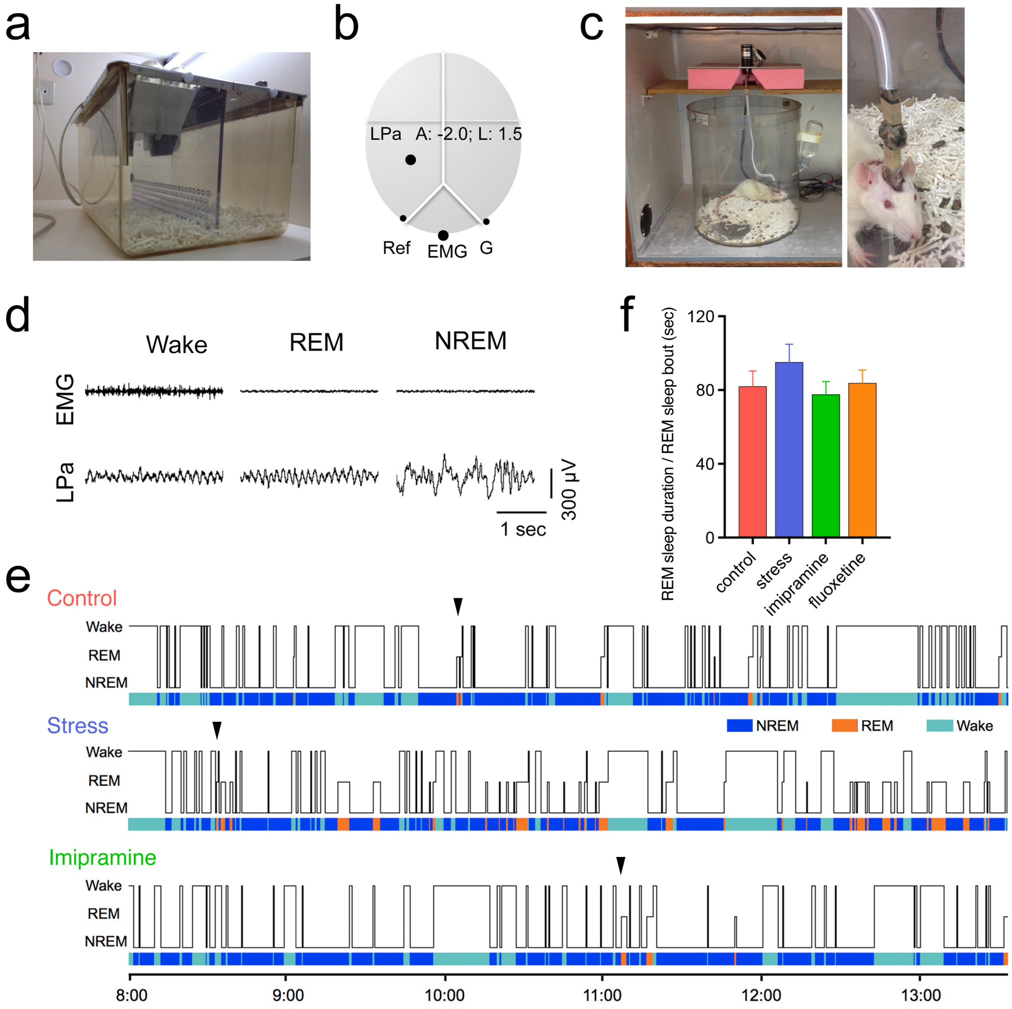

Supplementary Fig. S2 Cortical electroencephalography (EEG) recordings and offline analyses. (**a**) The experimental cage used for indirect contact during SDS. (**b**) The position of screw electrodes. (**c**) A free-moving rat with electrodes being measured by EEG and electromyography (EMG) in a cylinder (left). The recording cable has a voltage follower to reduce artifacts (right). (**d**) Typical EEG (LPa) and EMG recordings of the WAKE, REM, and NREM stages. (**e**) Representative hypnograms during the light phase (ZT0–ZT6). A black arrow linking three hypnograms (Control, Stress, and Imipramine) indicates the timing of the first rapid eye movement (REM) sleep (= REM sleep latency). (**f**) REM sleep bout duration.

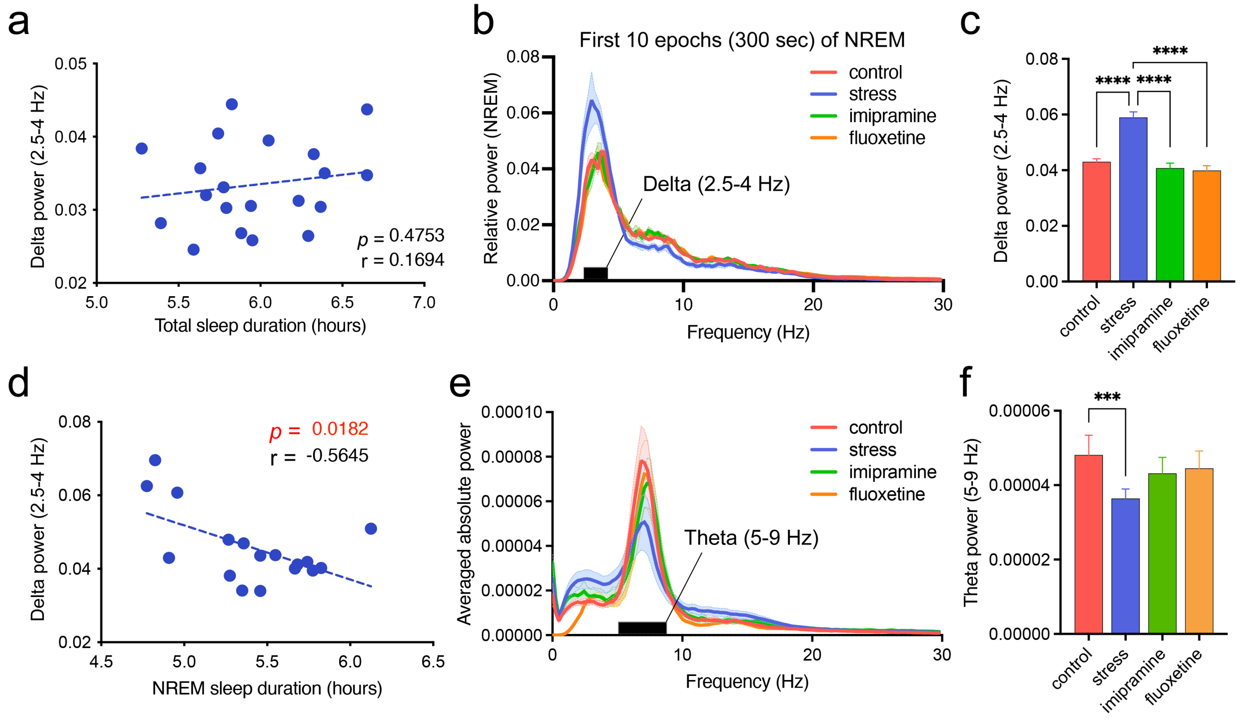

Supplementary Fig. S3 Effects of SDS and chronic AD treatment on EEG spectral power. (**a**) Correlations between total sleep duration and non-rapid eye movement (NREM) sleep delta power during the light phase (ZT0–ZT8.5) at 1 month after the last SDS. (**b**) EEG-relative power spectra during the first 10 epochs (total 300 sec) of NREM sleep in the light phase at 1 month after the last SDS. The black bar indicates the selected delta (2.5 – 4 Hz) band. (**c**) Average relative spectral power of the delta band during the first-10-epoch NREM sleep. $F_{(3, 78)} = 28.76$, $p < 0.0001$, ****$p < 0.0001$, two-way ANOVA followed by Tukey's multiple comparisons test. (**d**) Correlations between the first-10-epoch NREM sleep delta power and NREM sleep duration during the light phase at 1 month after the last SDS. $p = 0.0182$, $r = -0.5645$. (**e**) Absolute power spectra during REM sleep in the light phase 1 month after the last SDS. The black bar indicates the selected theta (5 – 9 Hz) band. For the absolute power analysis, EEG spectral data were excluded in one rat, due to artifacts that made processing unfeasible. (**f**) Average absolute spectral power of the theta band during REM sleep. $F_{(15, 256)} = 11.55$, $p < 0.0001$, ***$p < 0.001$, two-way ANOVA followed by Tukey's multiple comparisons test.

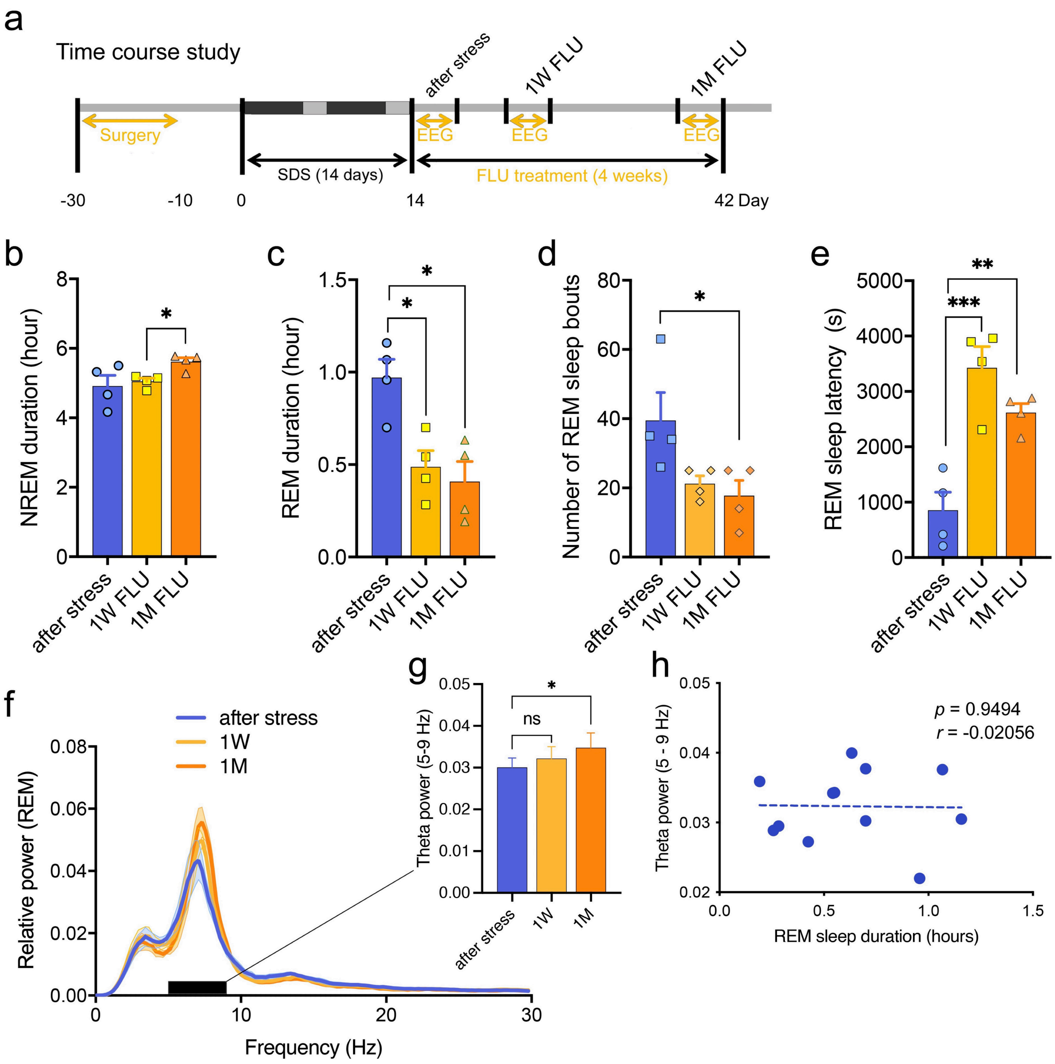

Supplementary Fig. S4 Effects of chronic fluoxetine (FLU) treatment on sleep architecture and REM sleep theta power. (**a**) Experimental schedule for sleep analysis. (**b**) Time-dependent effects of FLU on NREM duration in the light phase (ZT0–ZT8.5). The stressed rats were treated with FLU for 1 week (1W FLU) or 1 month (1M FLU), respectively. $F_{(2, 5)} = 7.068$, $p = 0.0308$, $*p < 0.05$, Welch's ANOVA test followed by Dunnett's multiple comparisons test. n = 4/group. (**c**) Time-dependent effects of FLU on REM duration. $F_{(2, 9)} = 9.486$, $p = 0.0064$,$*p < 0.05$, Brown-Forsythe ANOVA test followed by Tukey's test. n = 4/group. (**d**) Time-dependent effects of FLU on the number of REM sleep bouts. $F_{(2, 9)} = 4.544$, $p = 0.0432$,$*p < 0.05$, one-way ANOVA test followed by Tukey's test. n = 4/group. (**e**) Time-dependent effects of FLU on REM sleep latency. $F_{(2, 9)} = 18.44$, $p = 0.0007$, $**p < 0.01$, $***p < 0.001$, one-way ANOVA followed by Tukey's test. n = 4/group. (**f**) Time-dependent effects of FLU on the average relative spectral power during REM sleep. The black bar indicates selected theta (5–9 Hz) wavelengths. (**g**) Time-dependent effects of FLU on the average relative spectral powers of the theta band during REM sleep. $F_{(15, 144)} = 18.28$, $p < 0.0001$, $*p < 0.05$, two-way ANOVA followed by Tukey's multiple comparisons test. (**h**) Correlations between total REM sleep durations and REM sleep theta powers. The dots represent all the data from the three conditions (after stress, 1W FLU, and 1M FLU).

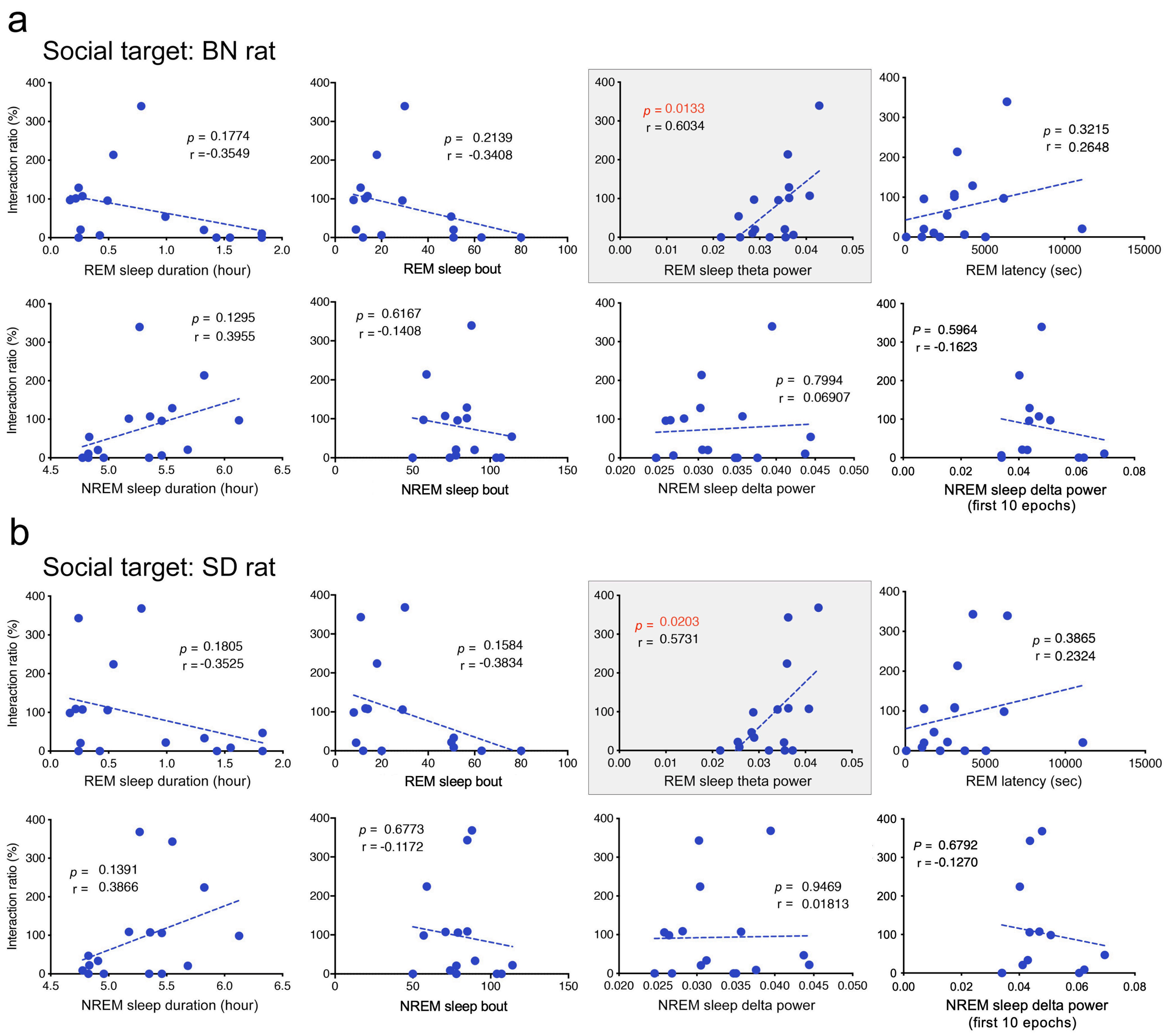

Supplementary Fig. S5 Supplementary data for Fig. 5. (**a**) Correlations between sleep variables at 1 month and interaction ratios toward BN rats at 1 month. Note that only REM sleep theta power correlated significantly with the interaction ratio toward BN rats. The dots represent the data from all the experimental rats (total 16 SD rats including control (n = 5), stressed (n = 6), and stressed rats with imipramine (IMI) treatments (n = 5)). The gray-colored graph is the same as Fig. 5b. (**b**) Correlations between sleep variables and interaction ratios toward unfamiliar SD rats. Note that only REM sleep theta power correlated significantly with the interaction ratio toward unfamiliar SD rats. The dots represent the data from all the experimental rats (total 16 SD rats including control (n = 5), stressed (n = 6), and stressed rats with IMI treatments (n = 5)). The gray-colored graph is the same as Fig. 5c.
