# Supplementary material for: Chronic antidepressant treatment rescues abnormally reduced REM sleep theta power in socially defeated rats
Source: Sci Rep. 2021 Aug 18;11:16713. doi: 10.1038/s41598-021-96094-0 (PMC8373914; doi:10.1038/s41598-021-96094-0)
Supplement: Supplementary file 2 — Supplementary Information 2. [file 41598_2021_96094_MOESM2_ESM.pdf]

Chronic antidepressant treatment rescues abnormally reduced REM sleep theta power in socially defeated rats

Yoshiki Matsuda, Ph.D.[1] , Nobuyuki Ozawa, Ph.D.[1], Takiko Shinozaki, B.D.[1], Kazuhisa Aoki, Ph.D.[1], Naomi Nihonmatsu-Kikuchi, Ph.D.[1], Toshikazu Shinba, M.D., Ph.D.[1, 2], Yoshitaka Tatebayashi, M.D., Ph.D.[1]
[1] *Affective Disorders Research Project, Tokyo Metropolitan Institute of Medical Science, Tokyo, JAPAN*
[2] *Department of Psychiatry, Shizuoka Saiseikai General Hospital, Shizuoka, JAPAN.*

Supplementary Table S1. Summary of the sleep changes in the chronic stress models. The information in the colored cells indicates the post-stress effects of chronic stress.

Chronic stress Model	Species	Duration of stress	Methods	EEG timing	REM sleep duration	REM sleep bout number	REM theta power	REM latency	NREM sleep duration	NREM sleep bout number	NREM delta power	Post-stress abnormalities	Effects of antidepressants	Reference	Comments
Unpredictable chronic mild stress	Male albino rats	3 weeks		during stress	increased	N/A	N/A	shortened	N/A	N/A	N/A		N/A	Moreau et al., 1995[1]	
				soon after the end of the stress	returned to normal	N/A	N/A	returned to normal	N/A	N/A	N/A	quickly returned to normal			
Unpredictable chronic mild stress	Male Lister hooded rats	5 weeks		during stress	increased at 3rd week	probably increased	N/A	shortened maximally at 3rd week, then returned to normal at 5th week	decreased at 3rd week	probably increased	N/A		N/A	Cheeta et al., 1997[2]	
Unpredictable chronic mild stress	Male SD rats	5 weeks		4th week during the stress	increased	increased	N/A	no change	SWS1: increased SWS2: no change	SWS1: increased SWS2: no change	N/A		N/A	Grønli et al., 2004[3]	
Unpredictable chronic mild stress	Male BALB/cJ mice	9 weeks		during stress	increased	increased	increased	N/A	unchanged	increased	unchanged		N/A	Nollet et al., 2019[4]	
chronic immobilization stress	Male Wistar rats	10 days	daily 2hour immobilization stress	7th, 14th, and 21st day after the stress	devided into two groups (increased or decreased REM durations)	no change	decreased or no change	N/A	no change	N/A	N/A	devided into two groups (increased or decreased REM durations) and prolonged up to 21st day	N/A	Hegde et al., 2011[5]	
Water Immersion and Restraint Stress	Male C57Bl/6J mice	3 weeks	2 hour immobilization in the tube immersed in the water to the xiphoid process, Once a day for 6 consecutive days per week for 3 weeks	1st, 2nd, and 3rd week during the stress	increased	increased	decreased	no change	increased	no change	increased		N/A	Yasugaki et al., 2019[6]	
chronic SDS (Miczek-type*)	Male and female Fischer 344 rats	4 weeks	up to 5 (for male) and 10 (for female) times attacks and 1 hour indirect interaction, 3-4 sessions per week for 4 weeks	2nd week of the 4 week SDS**	decreased	decreased	N/A	N/A	no change	no change	N/A		N/A	Page et al., 2016[7]	*The SDS method was originally developed by Miczek (1979)[8]. **EEG was measured during the weekend during which no SDS was performed.
				One day after the end of the 4 week SDS**	returned to normal	partially returned to normal	N/A	N/A	no change	no change	N/A	Most of the parameters returned to the baseline levels			
chronic SDS (Miczek-type*)	Male C57Bl/6J mice	10 days	up to 5 min direct interaction and up to 30 min indirect interaction during the first 3 hours of the light phase	1st, 3rd, and 10th day of the 10-day SDS, after SDS sessions, EEG was subsequently measured***	decreased in light phase but increased in dark phase	decreased in light phase but increased in dark phase	decreased in light phase but unchaged in dark phase	N/A	unchanged in light phase but increased in dark phase	no change	increased in light phase but unchanged in dark phase		N/A	Henderson et al., 2017[9]	*The SDS method was originally developed by Miczek (1979)[8]. ***EEG was measured subsequently after the SDS, thus the SDS directly affect the EEG data.
				5 days after the last stress	dcreased in the first 3 hours of the light phase only in the susceptible mice	N/A	no change	N/A	N/A	N/A	no change in the light phase	Slight changes were found only in the susceptible mice, Most of the parameters returned to the baseline levels			
chronic SDS (Miczek-type*)	Male SD rats	7 days	3-15 min direct interaction and up to 30 min indirect interaction during the first 3 hours of the light phase	1st and 7th day of the SDS session***	no change	no change	N/A	N/A	decreased in the passive coping group	N/A	N/A		N/A	Grafe et al., 2020[10]	*The SDS method was originally developed by Miczek (1979)[8]. ***EEG was measured subsequently after the SDS, thus the SDS directly affect the EEG data.
				2 weeks after the last SDS	no change	no change	N/A	N/A	decreased in the passive coping group	N/A	N/A	devided into passive and active coping groups, decreased SWS time only in the passive coping group			
chronic SDS (Berton-Krishnan-type)	Male C57Bl/6J mice	10 days	10 min direct interaction & 24 hour indirect interaction for 10 consecutive days	during 10 day SDS	increased	increased	increased at day1 but returned to control levels at day10	N/A	increased	no change	increased at day1 but returned to control levels at day10		N/A	Wells et al., 2017[11]	
				5 days after the last SDS	returned to control levels	increased	N/A	N/A	returned to control levels	no change	N/A	Most of the parameters, except for REM sleep bout number, returned to the baseline levels			
chronic SDS (Berton-Krishnan-type)	Male C57Bl/6J mice	10 days	10 min direct interaction & 24 hour indirect interaction for 10 consecutive days	3 day after the SDS	no change	no change	N/A	N/A	no change	no change	no change, but subsequent sleep deprivation increases NREM delta powers only in stressed mice	Vigilance states did not differ between stressed and control mice during post-stress baseline, sleep deprivation or recovery	N/A	Olini et al., 2017[12]	
chronic SDS (Berton-Krishnan-type)	Male SD rats	14 days	10 min direct interaction & 24 hour indirect interaction for 5 consecutive days plus 48 hour indirect interaction during the weekend for total 2 weeks	**18-28 days after the last SDS**	**increased**	**increased**	**decreased**	**shortened**	**decreased**	**increased**	**increased**	**Changes were prolonged up to one month**	**rescued**	Matsuda et al., present study	

N/A: not available

References

1. Moreau, J. L., Scherschlicht, R., Jenck, F., Martin, J. R. Chronic mild stress-induced anhedonia model of depression; sleep abnormalities and curative effects of electroshock treatment. Behav. Pharmacol. **6**, 682-687 (1995).
2. Cheeta, S., Ruigt, G., van Proosdij, J., Willner, P. Changes in sleep architecture following chronic mild stress. Biol. Psychiatry **41**, 419–427 (1997).
3. Grønli, J., Murison, R., Bjorvatn, B., Sørensen, E., Portas, C. M., Ursin, R. Chronic mild stress affects sucrose intake and sleep in rats. Behav. Brain Res. **150**, 139-47 (2004).
4. Nollet, M., Hicks, H., McCarthy, A. P., Wu, H., Möller-Levet, C. S., Laing, E. E., Malki, K., Lawless, N., Wafford, K. A., Dijk, D. J., Winsky-Sommerer, R. REM sleep's unique associations with corticosterone regulation, apoptotic pathways, and behavior in chronic stress in mice. Proc. Natl. Acad. Sci. USA **116**, 2733-2742 (2019).
5. Hegde, P., Jayakrishnan, H. R., Chattarji, S., Kutty, B. M. & Laxmi, T. R. Chronic stress-induced changes in rem sleep on θ oscillations in the rat hippocampus and amygdala. Brain Res. **1382**, 155-164 (2011).
6. Yasugaki, S., Liu, C. Y., Kashiwagi, M., Kanuka, M., Honda, T., Miyata, S., Yanagisawa, M., Hayashi, Y. Effects of 3 weeks of water immersion and restraint stress on sleep in mice. Front. Neurosci. **13**, 1072 (2019).
7. Page, G. G., Opp, M. R., Kozachik, S. L. Sex differences in sleep, anhedonia, and HPA axis activity in a rat model of chronic social defeat. Neurobiol. Stress **3**, 105–113 (2016).
8. Miczek, K. A. A new test for aggression in rats without aversive stimulation: differential effects of d-amphetamine and cocaine. Psychopharmacology **60**, 253–259 (1979).
9. Henderson, F., Vialou, V., Mestikawy, S. E., Fabre, V. Effects of social defeat stress on sleep in mice. Front. Behav. Neurosci. **11**, 227 (2017).
10. Grafe, LA, O'Mara, L, Branch, A, Dobkin, J, Luz, S, Vigderman, A, Shingala, A, Kubin, L, Ross, R, Bhatnagar, S. Passive coping strategies during repeated social defeat are associated with long-lasting changes in sleep in rats. Front. Syst. Neurosci. **14**, 6 (2020).
11. Wells, A. M., Ridener, E., Bourbonais, C. A., Kim, W., Pantazopoulos, H., Carroll, F. I., Kim, K. S., Cohen, B. M., Carlezon, W. A., Jr. Effects of chronic social defeat stress on sleep and circadian rhythms are mitigated by kappa-opioid receptor antagonism. J. Neurosci. **37**, 7656-7668 (2017).
12. Olini, N., Rothfuchs, I., Azzinnari, D., Pryce, C., Kurth, S., Huber, R. Chronic social stress leads to altered sleep homeostasis in mice. Behav. Brain Res. **327**, 167-173 (2017).
